# (*E*)-2-{[2-(2-Hy­droxy­ethyl­amino)­ethyl­imino]­meth­yl}phenol

**DOI:** 10.1107/S1600536811039997

**Published:** 2011-10-05

**Authors:** Juan M. Germán-Acacio, Hugo Tlahuext, Herbert Höpfl

**Affiliations:** a Centro de Investigaciones Químicas, Universidad Autónoma del Estado de Morelos. Av. Universidad 1001, CP 62209, Cuernavaca, Mexico

## Abstract

The asymmetric unit of the title compound, C_11_H_16_N_2_O_2_, contains two independent conformational isomers which show intra­molecular aromatic–imine O—H⋯N hydrogen bonds. In the crystal, neighboring mol­ecules are linked through inter­molecular aliphatic–aliphatic O—H⋯N, aliphatic–aromatic N—H⋯O and C—H⋯O inter­actions into hydrogen-bonded layers parallel to the *ab* plane.

## Related literature

For crystal structures of metal complexes with this ligand, see: Haber *et al.* (2003 ▶); Kenar *et al.* (2001 ▶); Li *et al.* (1988 ▶); Rajendiran *et al.* (2007 ▶). For supra­molecular assemblies with structurally related ligands, see: Barba *et al.* (2000 ▶); Fujita *et al.* (2008 ▶); Höpfl (2002 ▶); Severin (2009 ▶). For the tautomerism of salicyl­idene­imines, see: Domínguez *et al.* (2011 ▶); Fujiwara *et al.* (2009 ▶); Ogawa *et al.* (1998 ▶); Rodríguez *et al.* (2007 ▶).
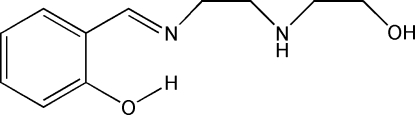


## Experimental

### 

#### Crystal data

C_11_H_16_N_2_O_2_
*M* *_r_* = 208.26
Orthorhombic, 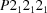

*a* = 7.0047 (11) Å
*b* = 14.171 (2) Å
*c* = 21.681 (3) Å
*V* = 2152.1 (6) Å^3^
*Z* = 8
Mo *K*α radiation
μ = 0.09 mm^−1^
*T* = 100 K
0.45 × 0.08 × 0.07 mm

#### Data collection

Bruker SMART CCD area-detector diffractometer
Absorption correction: multi-scan (*SADABS*; Sheldrick, 2003 ▶) *T*_min_ = 0.786, *T*_max_ = 0.994
12054 measured reflections
2677 independent reflections
2311 reflections with *I* > 2σ(*I*)
*R*_int_ = 0.052

#### Refinement

*R*[*F* ^2^ > 2σ(*F* ^2^)] = 0.052
*wR*(*F* ^2^) = 0.115
*S* = 1.14
2677 reflections
289 parameters
6 restraints
H atoms treated by a mixture of independent and constrained refinement
Δρ_max_ = 0.26 e Å^−3^
Δρ_min_ = −0.24 e Å^−3^

### 

Data collection: *SMART* (Bruker, 2000 ▶); cell refinement: *SAINT-Plus* (Bruker, 2001 ▶); data reduction: *SAINT-Plus*; program(s) used to solve structure: *SHELXS97* (Sheldrick, 2008 ▶); program(s) used to refine structure: *SHELXL97* (Sheldrick, 2008 ▶); molecular graphics: *SHELXTL* (Sheldrick, 2008 ▶) and *DIAMOND* (Brandenburg, 2006 ▶); software used to prepare material for publication: *SHELXTL* and *PLATON* (Spek, 2009 ▶).

## Supplementary Material

Crystal structure: contains datablock(s) I, global. DOI: 10.1107/S1600536811039997/pk2347sup1.cif

Structure factors: contains datablock(s) I. DOI: 10.1107/S1600536811039997/pk2347Isup2.hkl

Supplementary material file. DOI: 10.1107/S1600536811039997/pk2347Isup3.cml

Additional supplementary materials:  crystallographic information; 3D view; checkCIF report

## Figures and Tables

**Table 1:** Hydrogen-bond geometry (Å, °)

*D*—H⋯*A*	*D*—H	H⋯*A*	*D*⋯*A*	*D*—H⋯*A*
O1—H1⋯N1	0.84 (2)	1.77 (2)	2.562 (3)	157 (3)
O2—H2⋯N32^i^	0.84 (1)	2.00 (1)	2.839 (3)	178 (4)
N2—H2*A*⋯O1^i^	0.86 (2)	2.27 (2)	3.106 (3)	165 (2)
O31—H31⋯N31	0.84 (2)	1.82 (2)	2.596 (3)	154 (3)
O32—H32⋯N2^ii^	0.84 (1)	2.00 (1)	2.795 (3)	158 (4)
N32—H32*A*⋯O31^i^	0.86 (2)	2.55 (3)	3.347 (3)	154 (2)
C39—H39*A*⋯O32^iii^	0.99	2.49	3.443 (4)	161

Symmetry codes: (i) 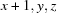; (ii) 
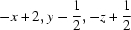
; (iii) 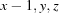.

**Fig. 1.**
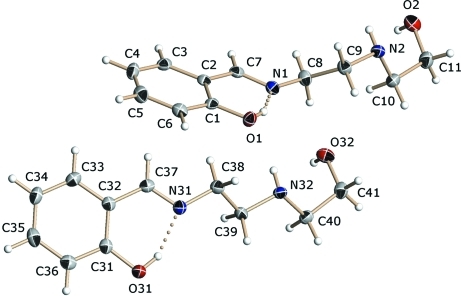
Perspective view of the asymmetric unit of (I). Displacement ellipsoids are drawn at the 50% probability level and H atoms are shown as small spheres of arbitrary radii.

**Fig. 2.**
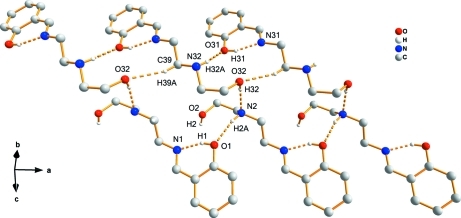
Fragment of the 2D layer parallel to the ab plane, showing intramolecular O–H···N and intermolecular N–H···O, O–H···N and C–H···O hydrogen bonding interactions. These fragments are linked further through O2–H2···N32 hydrogen bonds to give the overall 2D layer. Hydrogen atoms not involved in hydrogen bonding have been omitted for clarity.

## supplementary crystallographic information

### Comment

The title compound (I) has been employed as a tri- and tetradentate ligand for the complexation of transition metal ions such as vanadium(IV), vanadium(V), copper(II) and cadmium(II) (Haber *et al.*, 2003; Kenar *et al.*, 2001; Li *et al.*, 1988; Rajendiran *et al.*, 2007). We are interested in this compound in the search for ligands capable of forming macrocyclic structures with boronic acids (Barba *et al.*, 2000; Fujita *et al.*, 2008; Höpfl, 2002; Severin, 2009).

The asymmetric unit of (I) contains two conformers (Ia, Ib) (Fig. 1) with similar bond lengths between equivalent non-H atoms (differences less than 3 s.u.). The torsion angles (C–C–N–C) in the fragments CH_2_CH_2_NHCH_2 _are +62.7 (3) and -177.5 (2)° for Ia and Ib, respectively, showing that Ia and Ib are conformational isomers.  The dihedral angles in the NCH_2_CH_2_N ethylene fragments are -171.8 (2) and -176.1 (2)°, respectively.

From reports in the literature it is known that salicylidene imines form *keto* and *enol* tautomers, both in solution and the solid state (Domínguez *et al.*, 2011; Fujiwara *et al.*, 2009; Ogawa *et al.*, 1998; Rodríguez *et al.*, 2007). In the crystal structure both conformers correspond to the *enol* form.  This is evidenced by the presence of an intramolecular O–H···N hydrogen bond formed between the phenolic OH group and the imine function, and a comparative analysis of the bond lengths within the salicylidene fragment.  The values for the C_arom_–O and C=N bond lengths with values of 1.349 (3) and 1.274 (3)–1.275 (4) Å, respectively, are within the range expected for *enol* tautomers. The same is true for the C_arom_–C_arom_ bond lengths with values ranging from 1.374 (4)–1.417 (4) Å (Domínguez *et al.*, 2011). From a careful crystallographic analysis of eight salicylidene aminoalcohols, Domínguez *et al.* concluded that the dominant hydrogen bonding pattern in the crystal structures of *enol* tautomers are intermolecular O–H···O interactions formed between the pendant NCH_2_CH_2_OH fragments of neighboring molecules.  For keto tautomers, intermolecular O–H···O interactions typically involve the C_arom_–O oxygen atom (Domínguez *et al.*, 2011). In the crystal structure of the compound described herein, the crystallographically independent conformers are linked through O–H···N interactions between the pendant aliphatic NCH_2_CH_2_OH groups to give one-dimensional chains along axis *b*. Neighboring chains are further linked parallel to the *a* axis by N–H···O and C–H···O interactions involving as acceptor atoms the oxygen atoms of the phenolic and aliphatic O–H functions to form overall two-dimensional hydrogen bonded layers propagating parallel to the *ab* plane (Fig. 2).

### Experimental

For the preparation of (I), salicylaldehyde (0.234 g, 1.92 mmol) and 2-(2-aminoethylamino)ethanol (0.200 g, 1.92 mmol) were dissolved in 20 ml of ethanol. After reflux for 1 h, 60 ml of chloroform were added and the resulting solution was dried over anhydrous MgSO_4_. Evaporation of the solvent mixture under vacuum gave a yellow oil. Crystals suitable for X-ray diffraction analysis were grown from a solution in chloroform, which was overlayered with *n*-hexane. Yield: 0.310 g (77%). M.p. 358 K.

### Refinement

H atoms were positioned geometrically and constrained using the riding-model approximation [*C*–H_aryl_ and *C*–H_imine_ = 0.95 Å, *C*–H_aliphatic_ = 0.99 Å, *U*_iso_(H) = 1.2 *U*_eq_(C)]. Hydrogen atoms bonded to O and N were located in difference Fourier maps; however, the coordinates of the O–H and N–H hydrogen atoms were refined with distance and isotropic displacement parameter restraints: O–H = 0.840 (1) Å, N–H = 0.860 (1) Å and [*U*_iso_(H) = 1.5 *U*_eq_(O, N)].

### Crystal data

**Table:**

C_11_H_16_N_2_O_2_	*D*_x_ = 1.286 Mg m^−^^3^
*M**_r_* = 208.26	Melting point: 358 K
Orthorhombic, *P*2_1_2_1_2_1_	Mo *K*α radiation, λ = 0.71073 Å
Hall symbol: P 2ac 2ab	Cell parameters from 2547 reflections
*a* = 7.0047 (11) Å	θ = 2.9–25.4°
*b* = 14.171 (2) Å	µ = 0.09 mm^−^^1^
*c* = 21.681 (3) Å	*T* = 100 K
*V* = 2152.1 (6) Å^3^	Plate, yellow
*Z* = 8	0.45 × 0.08 × 0.07 mm
*F*(000) = 896	

### Data collection

**Table:**

Bruker SMART CCD area-detector diffractometer	2677 independent reflections
Radiation source: fine-focus sealed tube	2311 reflections with *I* > 2σ(*I*)
graphite	*R*_int_ = 0.052
φ and ω scans	θ_max_ = 27.0°, θ_min_ = 1.7°
Absorption correction: multi-scan (*SADABS*; Sheldrick, 2003)	*h* = −8→8
*T*_min_ = 0.786, *T*_max_ = 0.994	*k* = −9→18
12054 measured reflections	*l* = −27→24

### Refinement

**Table:**

Refinement on *F*^2^	Primary atom site location: structure-invariant direct methods
Least-squares matrix: full	Secondary atom site location: difference Fourier map
*R*[*F*^2^ > 2σ(*F*^2^)] = 0.052	Hydrogen site location: inferred from neighbouring sites
*wR*(*F*^2^) = 0.115	H atoms treated by a mixture of independent and constrained refinement
*S* = 1.14	*w* = 1/[σ^2^(*F*_o_^2^) + (0.0493*P*)^2^ + 0.4481*P*] where *P* = (*F*_o_^2^ + 2*F*_c_^2^)/3
2677 reflections	(Δ/σ)_max_ < 0.001
289 parameters	Δρ_max_ = 0.26 e Å^−^^3^
6 restraints	Δρ_min_ = −0.24 e Å^−^^3^

### Special details

**Table:**

Geometry. All e.s.d.'s (except the e.s.d. in the dihedral angle between two l.s. planes) are estimated using the full covariance matrix. The cell e.s.d.'s are taken into account individually in the estimation of e.s.d.'s in distances, angles and torsion angles; correlations between e.s.d.'s in cell parameters are only used when they are defined by crystal symmetry. An approximate (isotropic) treatment of cell e.s.d.'s is used for estimating e.s.d.'s involving l.s. planes.
Refinement. Refinement of *F*^2^ against ALL reflections. The weighted *R*-factor *wR* and goodness of fit *S* are based on *F*^2^, conventional *R*-factors *R* are based on *F*, with *F* set to zero for negative *F*^2^. The threshold expression of *F*^2^ > σ(*F*^2^) is used only for calculating *R*-factors(gt) *etc*. and is not relevant to the choice of reflections for refinement. *R*-factors based on *F*^2^ are statistically about twice as large as those based on *F*, and *R*- factors based on ALL data will be even larger.

### Fractional atomic coordinates and isotropic or equivalent isotropic displacement parameters (Å^2^)

**Table:**

	*x*	*y*	*z*	*U*_iso_*/*U*_eq_	
O1	0.3350 (3)	0.69081 (15)	0.11061 (9)	0.0234 (5)	
H1	0.442 (2)	0.674 (2)	0.1244 (14)	0.035*	
O2	1.4419 (3)	0.62189 (16)	0.25568 (10)	0.0298 (5)	
H2	1.453 (6)	0.5647 (7)	0.2462 (17)	0.045*	
N1	0.6713 (3)	0.62184 (17)	0.12202 (11)	0.0210 (5)	
N2	1.0729 (3)	0.69806 (18)	0.22607 (11)	0.0215 (6)	
H2A	1.161 (3)	0.691 (2)	0.1990 (11)	0.032*	
C1	0.3377 (4)	0.6585 (2)	0.05212 (13)	0.0193 (6)	
C2	0.5001 (4)	0.61174 (19)	0.02788 (13)	0.0191 (6)	
C3	0.4962 (4)	0.5809 (2)	−0.03349 (13)	0.0224 (6)	
H3	0.6044	0.5495	−0.0502	0.027*	
C4	0.3373 (5)	0.5954 (2)	−0.07020 (14)	0.0273 (7)	
H4	0.3367	0.5744	−0.1118	0.033*	
C5	0.1788 (5)	0.6408 (2)	−0.04570 (14)	0.0280 (7)	
H5	0.0692	0.6505	−0.0707	0.034*	
C6	0.1789 (4)	0.6719 (2)	0.01469 (14)	0.0231 (6)	
H6	0.0693	0.7029	0.0308	0.028*	
C7	0.6692 (4)	0.59821 (19)	0.06535 (13)	0.0194 (6)	
H7	0.7802	0.5713	0.0474	0.023*	
C8	0.8463 (4)	0.6083 (2)	0.15798 (13)	0.0232 (6)	
H8A	0.9544	0.5946	0.1300	0.028*	
H8B	0.8307	0.5540	0.1863	0.028*	
C9	0.8875 (4)	0.6970 (2)	0.19473 (14)	0.0236 (7)	
H9A	0.8811	0.7519	0.1665	0.028*	
H9B	0.7858	0.7050	0.2260	0.028*	
C10	1.0972 (4)	0.6245 (2)	0.27303 (13)	0.0251 (7)	
H10A	1.0782	0.5616	0.2542	0.030*	
H10B	1.0007	0.6327	0.3060	0.030*	
C11	1.2954 (4)	0.6306 (2)	0.30039 (14)	0.0289 (7)	
H11A	1.3094	0.6920	0.3218	0.035*	
H11B	1.3106	0.5801	0.3315	0.035*	
O31	−0.2420 (3)	0.35742 (16)	0.10565 (9)	0.0252 (5)	
H31	−0.134 (2)	0.375 (2)	0.1175 (15)	0.038*	
O32	0.8773 (3)	0.39234 (15)	0.25880 (11)	0.0279 (5)	
H32	0.897 (5)	0.3345 (6)	0.2532 (17)	0.042*	
N31	0.1079 (3)	0.41785 (17)	0.10613 (11)	0.0207 (5)	
N32	0.4908 (3)	0.42869 (18)	0.22487 (11)	0.0196 (5)	
H32A	0.586 (3)	0.423 (2)	0.2002 (12)	0.029*	
C31	−0.2312 (4)	0.3602 (2)	0.04357 (13)	0.0204 (6)	
C32	−0.0647 (4)	0.3916 (2)	0.01297 (13)	0.0198 (6)	
C33	−0.0628 (4)	0.3926 (2)	−0.05144 (13)	0.0239 (7)	
H33	0.0479	0.4145	−0.0723	0.029*	
C34	−0.2171 (5)	0.3626 (2)	−0.08532 (14)	0.0272 (7)	
H34	−0.2124	0.3630	−0.1291	0.033*	
C35	−0.3797 (5)	0.3319 (2)	−0.05493 (15)	0.0284 (7)	
H35	−0.4868	0.3110	−0.0781	0.034*	
C36	−0.3872 (4)	0.3314 (2)	0.00889 (14)	0.0246 (7)	
H36	−0.5003	0.3111	0.0291	0.029*	
C37	0.1035 (4)	0.4199 (2)	0.04734 (13)	0.0203 (6)	
H37	0.2133	0.4406	0.0255	0.024*	
C38	0.2801 (4)	0.4497 (2)	0.13747 (13)	0.0219 (6)	
H38A	0.2631	0.5160	0.1508	0.026*	
H38B	0.3890	0.4476	0.1084	0.026*	
C39	0.3245 (4)	0.3893 (2)	0.19290 (13)	0.0208 (6)	
H39A	0.2133	0.3879	0.2211	0.025*	
H39B	0.3519	0.3238	0.1796	0.025*	
C40	0.5399 (4)	0.3768 (2)	0.28123 (13)	0.0228 (7)	
H40A	0.5419	0.3082	0.2726	0.027*	
H40B	0.4427	0.3889	0.3134	0.027*	
C41	0.7342 (4)	0.4083 (2)	0.30387 (15)	0.0255 (7)	
H41A	0.7297	0.4764	0.3140	0.031*	
H41B	0.7669	0.3734	0.3420	0.031*	

### Atomic displacement parameters (Å^2^)

**Table:**

	*U*^11^	*U*^22^	*U*^33^	*U*^12^	*U*^13^	*U*^23^
O1	0.0208 (10)	0.0298 (12)	0.0194 (11)	0.0035 (9)	0.0004 (9)	−0.0023 (9)
O2	0.0253 (12)	0.0260 (12)	0.0380 (13)	0.0013 (10)	−0.0014 (10)	−0.0016 (11)
N1	0.0205 (12)	0.0191 (13)	0.0233 (13)	−0.0006 (11)	−0.0006 (10)	−0.0003 (11)
N2	0.0187 (12)	0.0240 (13)	0.0218 (13)	−0.0014 (11)	0.0016 (10)	0.0007 (11)
C1	0.0243 (14)	0.0148 (14)	0.0188 (14)	−0.0034 (12)	0.0020 (12)	0.0043 (11)
C2	0.0226 (13)	0.0136 (14)	0.0210 (14)	−0.0054 (12)	0.0016 (12)	0.0030 (12)
C3	0.0273 (15)	0.0174 (15)	0.0224 (15)	−0.0026 (12)	0.0068 (12)	−0.0001 (12)
C4	0.0421 (18)	0.0217 (16)	0.0180 (15)	−0.0037 (15)	−0.0049 (14)	0.0001 (12)
C5	0.0304 (16)	0.0258 (17)	0.0277 (17)	0.0000 (14)	−0.0102 (14)	0.0052 (14)
C6	0.0210 (14)	0.0193 (15)	0.0290 (16)	0.0014 (12)	−0.0009 (13)	0.0027 (13)
C7	0.0182 (13)	0.0151 (14)	0.0250 (15)	−0.0013 (11)	0.0050 (12)	−0.0007 (12)
C8	0.0221 (14)	0.0221 (16)	0.0254 (16)	0.0017 (13)	−0.0048 (12)	−0.0011 (13)
C9	0.0195 (14)	0.0241 (16)	0.0272 (16)	0.0015 (13)	−0.0013 (12)	−0.0028 (14)
C10	0.0264 (15)	0.0296 (18)	0.0193 (15)	−0.0036 (14)	0.0017 (12)	0.0002 (14)
C11	0.0311 (17)	0.0310 (18)	0.0248 (16)	0.0002 (14)	−0.0048 (13)	0.0001 (14)
O31	0.0219 (10)	0.0321 (13)	0.0217 (11)	−0.0051 (10)	0.0009 (9)	0.0000 (10)
O32	0.0225 (11)	0.0244 (12)	0.0369 (13)	0.0006 (10)	0.0018 (9)	0.0042 (11)
N31	0.0215 (12)	0.0195 (13)	0.0211 (13)	0.0013 (10)	0.0001 (10)	−0.0016 (10)
N32	0.0183 (12)	0.0219 (13)	0.0187 (13)	−0.0010 (10)	0.0023 (10)	0.0022 (11)
C31	0.0242 (14)	0.0150 (15)	0.0221 (15)	0.0026 (12)	−0.0019 (12)	−0.0020 (12)
C32	0.0226 (13)	0.0140 (14)	0.0227 (15)	0.0013 (12)	−0.0012 (12)	0.0005 (12)
C33	0.0274 (15)	0.0230 (17)	0.0213 (15)	0.0040 (13)	0.0038 (12)	0.0020 (13)
C34	0.0412 (18)	0.0218 (16)	0.0186 (15)	0.0020 (14)	−0.0053 (13)	−0.0011 (13)
C35	0.0336 (18)	0.0197 (16)	0.0319 (17)	0.0010 (14)	−0.0132 (14)	−0.0015 (14)
C36	0.0276 (15)	0.0163 (15)	0.0299 (17)	0.0009 (12)	0.0011 (13)	0.0018 (13)
C37	0.0199 (14)	0.0184 (15)	0.0225 (15)	0.0030 (12)	0.0032 (12)	0.0011 (12)
C38	0.0209 (14)	0.0220 (16)	0.0227 (15)	−0.0006 (12)	−0.0002 (11)	0.0016 (13)
C39	0.0170 (12)	0.0227 (16)	0.0228 (14)	−0.0021 (13)	−0.0007 (12)	−0.0004 (13)
C40	0.0239 (15)	0.0252 (17)	0.0192 (15)	0.0000 (13)	0.0013 (12)	0.0007 (13)
C41	0.0293 (15)	0.0241 (17)	0.0230 (15)	0.0030 (14)	−0.0055 (13)	−0.0003 (13)

### Geometric parameters (Å, °)

**Table:**

O1—C1	1.349 (3)	O31—C31	1.349 (3)
O1—H1	0.8400 (11)	O31—H31	0.8401 (11)
O2—C11	1.417 (4)	O32—C41	1.418 (4)
O2—H2	0.8401 (11)	O32—H32	0.8400 (11)
N1—C7	1.274 (3)	N31—C37	1.275 (4)
N1—C8	1.466 (3)	N31—C38	1.456 (4)
N2—C9	1.466 (4)	N32—C39	1.466 (3)
N2—C10	1.467 (4)	N32—C40	1.467 (4)
N2—H2A	0.8600 (11)	N32—H32A	0.8600 (11)
C1—C6	1.391 (4)	C31—C36	1.388 (4)
C1—C2	1.417 (4)	C31—C32	1.413 (4)
C2—C3	1.401 (4)	C32—C33	1.397 (4)
C2—C7	1.448 (4)	C32—C37	1.451 (4)
C3—C4	1.383 (4)	C33—C34	1.374 (4)
C3—H3	0.9500	C33—H33	0.9500
C4—C5	1.389 (5)	C34—C35	1.386 (5)
C4—H4	0.9500	C34—H34	0.9500
C5—C6	1.382 (4)	C35—C36	1.385 (4)
C5—H5	0.9500	C35—H35	0.9500
C6—H6	0.9500	C36—H36	0.9500
C7—H7	0.9500	C37—H37	0.9500
C8—C9	1.516 (4)	C38—C39	1.508 (4)
C8—H8A	0.9900	C38—H38A	0.9900
C8—H8B	0.9900	C38—H38B	0.9900
C9—H9A	0.9900	C39—H39A	0.9900
C9—H9B	0.9900	C39—H39B	0.9900
C10—C11	1.512 (4)	C40—C41	1.514 (4)
C10—H10A	0.9900	C40—H40A	0.9900
C10—H10B	0.9900	C40—H40B	0.9900
C11—H11A	0.9900	C41—H41A	0.9900
C11—H11B	0.9900	C41—H41B	0.9900
			
C1—O1—H1	103 (2)	C31—O31—H31	104 (2)
C11—O2—H2	109 (3)	C41—O32—H32	112 (3)
C7—N1—C8	119.2 (3)	C37—N31—C38	118.6 (3)
C9—N2—C10	114.7 (2)	C39—N32—C40	112.9 (2)
C9—N2—H2A	109 (2)	C39—N32—H32A	107 (2)
C10—N2—H2A	108 (2)	C40—N32—H32A	107 (2)
O1—C1—C6	119.4 (3)	O31—C31—C36	119.2 (3)
O1—C1—C2	121.3 (3)	O31—C31—C32	121.6 (3)
C6—C1—C2	119.4 (3)	C36—C31—C32	119.2 (3)
C3—C2—C1	118.8 (3)	C33—C32—C31	118.7 (3)
C3—C2—C7	120.5 (3)	C33—C32—C37	120.2 (3)
C1—C2—C7	120.7 (2)	C31—C32—C37	121.1 (2)
C4—C3—C2	121.1 (3)	C34—C33—C32	121.6 (3)
C4—C3—H3	119.5	C34—C33—H33	119.2
C2—C3—H3	119.5	C32—C33—H33	119.2
C3—C4—C5	119.5 (3)	C33—C34—C35	119.3 (3)
C3—C4—H4	120.3	C33—C34—H34	120.4
C5—C4—H4	120.3	C35—C34—H34	120.4
C6—C5—C4	120.7 (3)	C36—C35—C34	120.5 (3)
C6—C5—H5	119.7	C36—C35—H35	119.7
C4—C5—H5	119.7	C34—C35—H35	119.7
C5—C6—C1	120.6 (3)	C35—C36—C31	120.6 (3)
C5—C6—H6	119.7	C35—C36—H36	119.7
C1—C6—H6	119.7	C31—C36—H36	119.7
N1—C7—C2	121.0 (3)	N31—C37—C32	121.8 (3)
N1—C7—H7	119.5	N31—C37—H37	119.1
C2—C7—H7	119.5	C32—C37—H37	119.1
N1—C8—C9	109.3 (2)	N31—C38—C39	111.5 (2)
N1—C8—H8A	109.8	N31—C38—H38A	109.3
C9—C8—H8A	109.8	C39—C38—H38A	109.3
N1—C8—H8B	109.8	N31—C38—H38B	109.3
C9—C8—H8B	109.8	C39—C38—H38B	109.3
H8A—C8—H8B	108.3	H38A—C38—H38B	108.0
N2—C9—C8	114.9 (2)	N32—C39—C38	108.9 (2)
N2—C9—H9A	108.5	N32—C39—H39A	109.9
C8—C9—H9A	108.5	C38—C39—H39A	109.9
N2—C9—H9B	108.5	N32—C39—H39B	109.9
C8—C9—H9B	108.5	C38—C39—H39B	109.9
H9A—C9—H9B	107.5	H39A—C39—H39B	108.3
N2—C10—C11	109.8 (3)	N32—C40—C41	109.5 (2)
N2—C10—H10A	109.7	N32—C40—H40A	109.8
C11—C10—H10A	109.7	C41—C40—H40A	109.8
N2—C10—H10B	109.7	N32—C40—H40B	109.8
C11—C10—H10B	109.7	C41—C40—H40B	109.8
H10A—C10—H10B	108.2	H40A—C40—H40B	108.2
O2—C11—C10	113.0 (2)	O32—C41—C40	111.4 (2)
O2—C11—H11A	109.0	O32—C41—H41A	109.3
C10—C11—H11A	109.0	C40—C41—H41A	109.3
O2—C11—H11B	109.0	O32—C41—H41B	109.3
C10—C11—H11B	109.0	C40—C41—H41B	109.3
H11A—C11—H11B	107.8	H41A—C41—H41B	108.0
			
O1—C1—C2—C3	−179.0 (3)	O31—C31—C32—C33	179.9 (3)
C6—C1—C2—C3	0.4 (4)	C36—C31—C32—C33	0.1 (4)
O1—C1—C2—C7	−0.6 (4)	O31—C31—C32—C37	1.5 (4)
C6—C1—C2—C7	178.8 (2)	C36—C31—C32—C37	−178.3 (3)
C1—C2—C3—C4	0.0 (4)	C31—C32—C33—C34	−1.0 (4)
C7—C2—C3—C4	−178.5 (3)	C37—C32—C33—C34	177.4 (3)
C2—C3—C4—C5	−0.4 (5)	C32—C33—C34—C35	0.9 (5)
C3—C4—C5—C6	0.4 (5)	C33—C34—C35—C36	0.1 (5)
C4—C5—C6—C1	0.0 (5)	C34—C35—C36—C31	−1.0 (5)
O1—C1—C6—C5	179.0 (3)	O31—C31—C36—C35	−178.9 (3)
C2—C1—C6—C5	−0.4 (4)	C32—C31—C36—C35	0.9 (4)
C8—N1—C7—C2	−179.0 (2)	C38—N31—C37—C32	−178.4 (2)
C3—C2—C7—N1	−176.6 (3)	C33—C32—C37—N31	−178.4 (3)
C1—C2—C7—N1	5.0 (4)	C31—C32—C37—N31	0.0 (4)
C7—N1—C8—C9	133.4 (3)	C37—N31—C38—C39	−141.5 (3)
C10—N2—C9—C8	−62.7 (3)	C40—N32—C39—C38	177.5 (2)
N1—C8—C9—N2	−171.8 (2)	N31—C38—C39—N32	−176.1 (2)
C9—N2—C10—C11	178.0 (2)	C39—N32—C40—C41	168.2 (2)
N2—C10—C11—O2	−57.8 (4)	N32—C40—C41—O32	−59.2 (3)

### Hydrogen-bond geometry (Å, °)

**Table:**

*D*—H···*A*	*D*—H	H···*A*	*D*···*A*	*D*—H···*A*
O1—H1···N1	0.84 (2)	1.77 (2)	2.562 (3)	157 (3)
O2—H2···N32^i^	0.84 (1)	2.00 (1)	2.839 (3)	178 (4)
N2—H2A···O1^i^	0.86 (2)	2.27 (2)	3.106 (3)	165 (2)
O31—H31···N31	0.84 (2)	1.82 (2)	2.596 (3)	154 (3)
O32—H32···N2^ii^	0.84 (1)	2.00 (1)	2.795 (3)	158 (4)
N32—H32A···O31^i^	0.86 (2)	2.55 (3)	3.347 (3)	154 (2)
C39—H39A···O32^iii^	0.99	2.49	3.443 (4)	161

Symmetry codes: (i) *x*+1, *y*, *z*; (ii) −*x*+2, *y*−1/2, −*z*+1/2; (iii) *x*−1, *y*, *z*.
